# Eosinophilic Cholecystitis presenting with Common Bile Duct Sludge and Cholangitis: A Case Report

**DOI:** 10.31729/jnma.4684

**Published:** 2020-03-31

**Authors:** Niraj Kumar Keyal, Pooja Adhikari, Basu Dev Baskota, Ujwal Rai, Aalok Thakur

**Affiliations:** 1 Department of Critical Care Medicine, B & C Medical College Teaching Hospital & Research Center, Birtamode, Jhapa, Nepal; 2 Department of Anaesthesiology & Critical Care, B & C Medical College Teaching Hospital & Research Center, Birtamode, Jhapa, Nepal; 3 Department of Surgery, B & C Medical College Teaching Hospital & Research Center, Birtamode, Jhapa, Nepal; 4 Department of Pathology, B & C Medical College Teaching Hospital & Research Center, Birtamode, Jhapa, Nepal

**Keywords:** *cholecystitis*, *eosinophilia*, *jaundice*, *obstructive*

## Abstract

Eosinophilic cholecystitis is a rare post-cholecystectomy inflammatory histopathological condition characterized by more than 90% eosinophilic infiltrate in the gallbladder. We present a case of 27-year female presented with abdominal pain, fever, jaundice, altered mental status, shock, leucocytosis, deranged liver function test, and peripheral blood eosinophilia. The patient underwent cholecystectomy and common bile duct exploration. She developed adult respiratory distress syndrome and hospital-acquired pneumonia. From this, we want to emphasize that eosinophilic cholecystitis and cholangiopathy should be a differential diagnosis in patients presenting with allergy, peripheral eosinophilia, obstructive jaundice that are planned to undergo cholecystectomy that will have early critical care intervention.

## INTRODUCTION

Eosinophilic cholecystitis (EC) is a rare form of cholecystitis that is diagnosed histopathologically; clinically it cannot be distinguished from other forms of cholecystitis.^1^ It commonly occurs in female at age of 25-64 years and incidence ranges from 0.25% to 6.4%.^1,2,3,4^ The aetiology is not known but EC is associated with allergies, hypereosinophilic syndrome, eosinophilic airway disease,^2^ lipomatosis, a parasitic infection like Clonorchiasis sinensis, hydatid cyst,^3^ drugs like cephalosporin, herbal medicines,^1^ cholangiopathy,^4^ gall stones,^5^ eosinophilic gastroenteritis,^6^ cystitides^7^ and autoimmune. Treatment of biliary cholangiopathy is conservative.^4^ This is the first case report of EC presenting as the common bile duct and gallbladder sludge.

## CASE REPORT

A 27 years old female, with a history of occasional itching and a skin rash that subsided itself presented to the emergency department with right upper quadrant abdominal pain, fever of 1020 Fahrenheit, jaundice, altered mental status for 2 days. At presentation in the Emergency department, Glasgow Coma Scale (GCS) was 15/15, pulse rate 110 beats/per min, blood pressure (BP) 70/40 mm Hg, respiratory rate 26 breaths/ min, oxygen saturation 96% on 10 liters of oxygen. The chest and cardiovascular examination were normal. Her abdominal examination showed tenderness at the right hypochondriac region. Murphy's sign was positive. Arterial blood gas (ABG) analysis showed metabolic and lactic acidosis. She was immediately resuscitated with fluid and noradrenaline and Piperacillin-Tazobactam and Levofloxacin. After resuscitation for 6 hours, BP was 100/60 mm Hg and Noradrenaline were stopped on the second day.

Her blood investigation profiles were Total leucocyte count (TLC) 14000/mm3, neutrophil 80%, lymphocyte 10% and eosinophil 10% and absolute eosinophil count 1200/ mm3, platelets 120000/mm3, hemoglobin (Hb) 9gm/dl, urea 58 mg/dl, creatinine 1.3 mg/dl, sodium and potassium normal. Total bilirubin was 8.6mg/ dl in which direct was 6.8mg/dl. Total protein was 5.9mg/dl in which albumin was found to be 3.1mg/dl. Alanine aminotransferase (ALT) was 601U/L, Aspartate aminotransferase (AST) 681U/L, Alkaline phosphatase (ALP) 1004 U/L and Prothrombin time (PT) 18 seconds, International normalized (INR) ratio 2.1.

**Figure 1:**
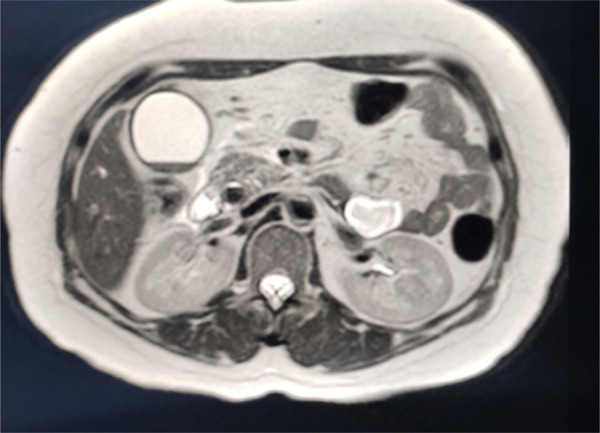
Magnetic resonance cholangiopancreatography showing gall stone, sludge or stone in Common Bile Duct dilation of the intrahepatic and extrahepatic biliary duct.

Ultrasound of the abdomen showed features of calculus cholecystitis with pericholecystic collection, thickened gallbladder wall and dilated Common Bile Duct (CBD). Magnetic resonance cholangiopancreatography showed gallstone with sludge or stone in CBD with dilation of intrahepatic and extrahepatic biliary duct (Figure 1).

**Figure 2:**
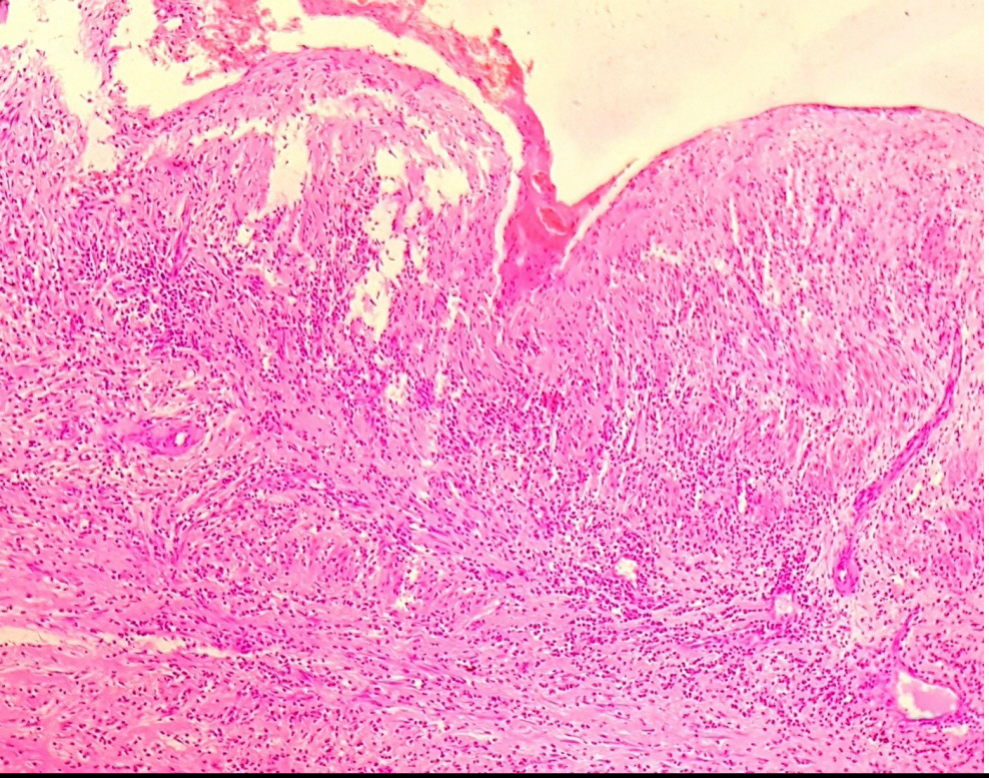
Hematoxylin and Eosin section showing sheets of eosinophils within mucosa and muscularis propria with edema 40x magnification.

The patient was diagnosed as choledocholithiasis with obstructive jaundice and was advised for Endoscopic Retrograde Cholangiopancreatography (ERCP) but this was not available at our center. The patient underwent open cholecystectomy, CBD exploration, and T-Tube. Gallbladder and CBD did not have any stone but only sludge was present that was misdiagnosed as stone in radiological examination. The gallbladder was sent for histopathological examination. Histopathological examination showed more than 90% eosinophil infiltrating mucosa and muscularis propria of gallbladder and was diagnosed as EC (Figure 2).

**Figure 3:**
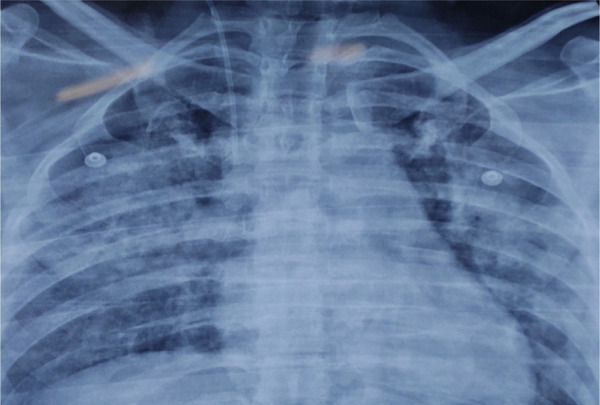
Chest X-ray showing bilateral infiltrates.

T-tube was removed after 16 days. The patient developed fever, shortness of breath, cough, abdominal distension, tachycardia and septic shock on the 18th day. Chest examination showed bilateral crepitation, x-ray bilateral infiltrates and ABG type 1 respiratory failure (Figure 3).

She was diagnosed as severe adult respiratory distress syndrome secondary to hospital-acquired pneumonia. The patient was intubated and was managed as per Intensive Care Unit (ICU) protocol. Tracheal aspirate showed Pseudomonas aeruginosa and Escherichia coli sensitive to Polymyxin B and Amikacin and Charcot Leden crystals. She was started on Polymyxin B 15 two times a day and Amikacin 750mg once a day. But, there was no response for 48 hours. Then, Caspofungin 50mg was started as her candid score was 4. The patient was extubated on 4th day and shifted out of ICU on the 7th day. She was discharged after 1 month of hospital stay. Antinuclear antibody test, stool examination, a peripheral blood smear was negative. Immunoglobulin assay was not done as it was not possible at our center. She was discharged on Cetirizine 5 mg daily for 2 weeks. She was followed up after 2 weeks and did not have any complaint and was asked to continue Cetirizine.

## DISCUSSION

EC is a rare, histopathological diagnosis without any specific sign, symptom, diagnostic and treatment modalities specific to it. Therefore mostly it is misdiagnosed and treated as acute calculus cholecystitis. Studies have shown that patients are diagnosed as gallstone and post cholecystectomy are diagnosed as EC that was seen in our patient. Peripheral eosinophilia may or may not be seen but is generally observed in 10-15% and represents the manifestation of hypereosinophilic syndrome.^8^

Our patient was having eosinophilia, urticaria, Charcot Leden crystal in sputum^9^ and occasional shortness of breath that was suggestive of hypereosinophilic syndrome which was missed. EC has no definite pathophysiology but has been associated with other disorders, peripheral eosinophilia and therefore, patient planned for cholecystectomy for any cause presenting with eosinophilia, allergic manifestation should be evaluated for EC.

A retrospective study was done on diagnosis, outcome and presenting symptoms of EC because it is generally diagnosed after cholecystectomy. Our patient presented with features of obstructive jaundice but any pathology obstructing bile flow at extrahepatic duct can present as obstructive jaundice. Therefore, it is a nonspecific presentation of EC. Treatment of eosinophilic cholangiopathy is conservative4 and ERCP is a treatment of choice for removal of stone from CBD but an exploration of CBD was done in our patient which might be the cause of complicated perioperative course in our patient as EC is a severe allergic inflammatory condition. Biliary tract disease is associated with eosinophilic gastroenteritis that was not seen in our patients. Therefore, large scale studies are required to know treatment options for EC that can decrease morbidity and mortality of the patient.

EC is a severe inflammatory condition that can cause mortality. Our patient had prolonged hospital stay and developed multidrug-resistant hospital-acquired pneumonia. The patient was started on antibiotics but had no response. Then the patient was treated with antifungal Caspofungin and underwent uneventful recovery. The candid score is a simple bedside score that helps to start empirically antifungal for invasive candidiasis because invasive candidiasis is undertreated and easily missed diagnosis that has very high morbidity and mortality.^10^ Therefore, a candid score should be calculated and antifungal should be started inthe patient not responding to the antibiotic.

To conclude, EC should be suspected in all patients presenting with gall stone; peripheral eosinophilia that is planned for cholecystectomy. Aggressive intensive care management is required for better patient outcomes.

## Consent:

**JNMA Case Report Consent Form** was signed by the patient and the original article is attached with the patient's chart.

## Conflict of Interest

**None.**
